# Supplementary material for: Enhanced mitophagy in bronchial fibroblasts from severe asthmatic patients
Source: PLoS One. 2020 Nov 30;15(11):e0242695. doi: 10.1371/journal.pone.0242695 (PMC7704010; doi:10.1371/journal.pone.0242695)

Figure 2 (Lanes highlighted in red are not included in this study)

Loading order: Ladder->NHBF->DHBF-> X->X

LC3B (Fig. 2C)

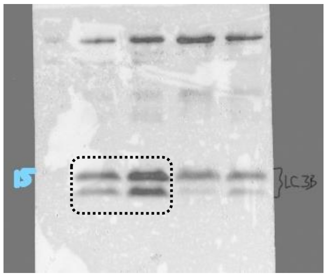

p62 (Fig. 2C)

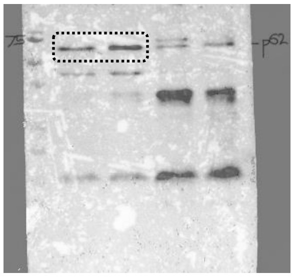

Loading order: Ladder->gap->X->X->X->X->NHBF->X->DHBF->X-> X->X->X->X

LAMP2A (Fig. 2C)

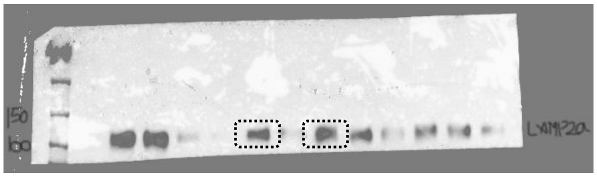

Loading order: Ladder-> X->X->X->X->NHBF->NHBF+E64d+PepA->DHBF->DHBF+E64d+PepA->X->X->X->X

LC3B (Fig. 2F)

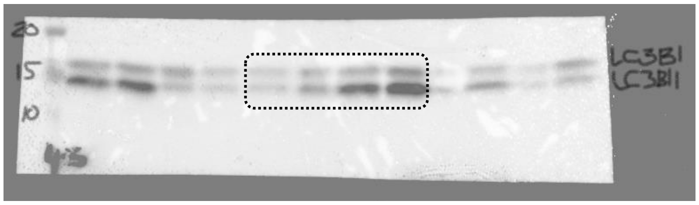

p62 (Fig. 2F)

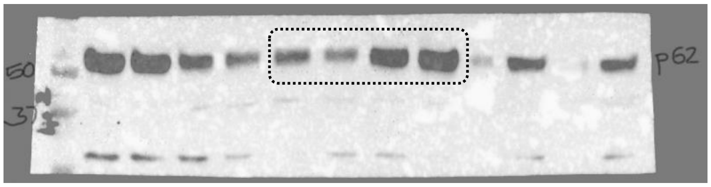

Figure 3

Loading order: Ladder-> NHBF (12h)-> NHBF (24h)->DHBF (12h)-> DHBF (24h)

PINK1 (Fig. 3B)

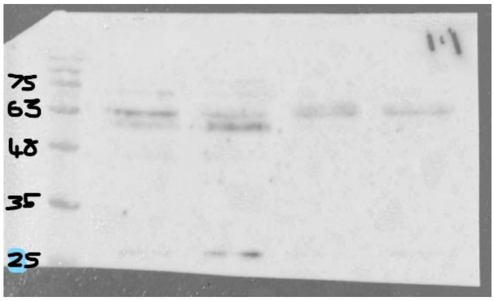

Loading order: Ladder-> NHBF->DHBF-> X->X

PINK1 (Fig. 3C)

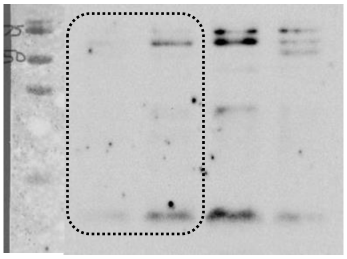

Parkin (Fig. 3C)

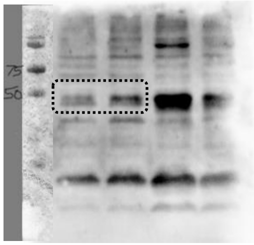

Optineurin & BNIP3 (Fig. 3C)

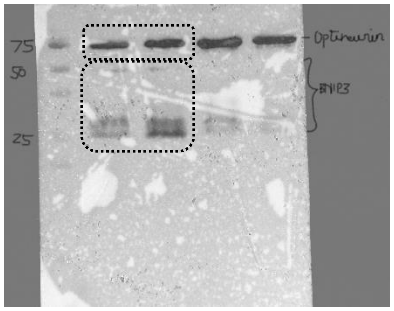

BNIP3L (Fig. 3C)

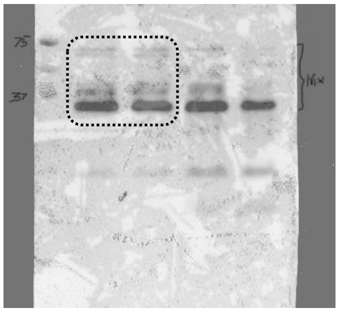

NDP52 (Fig. 3C)

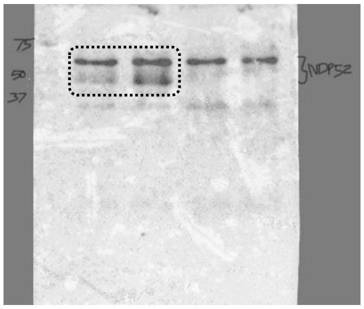

Loading order: Ladder-> X->X->X->X-> NHBF->NHBF+E64d+PepA->DHBF->DHBF+E64d+PepA->X->X->X->X

PINK1 (Fig. 3D)

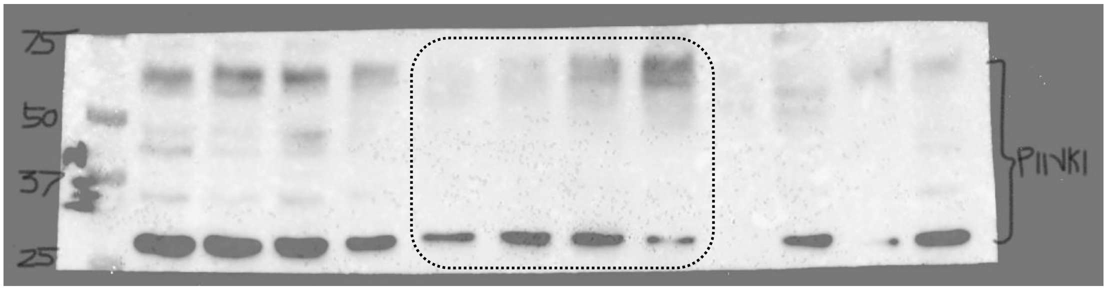

Figure 5

Loading order: Ladder->gap->NHBF->X->DHBF->X

p-AMPKα (Fig. 5A)

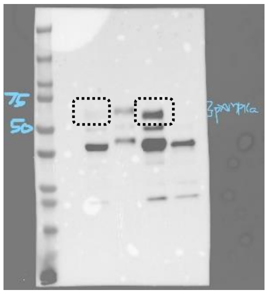

AMPKα (Fig. 5A)

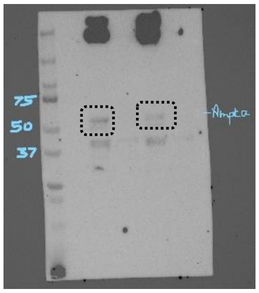

Loading order: X->X->X->NHBF->DHBF->X->X->X->X

SIRT1 (Fig. 5B)

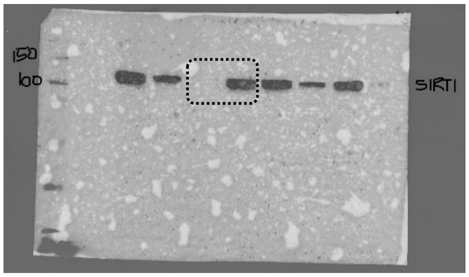

PGC1α (Fig. 5B)

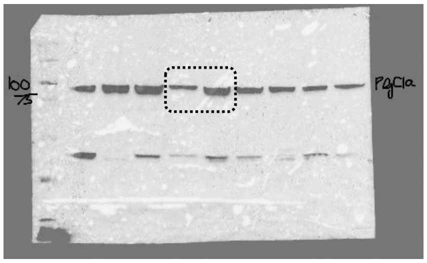

Supplement: S1 Raw images — (PDF) [file pone.0242695.s001.pdf]
