# Supplementary material for: Enhanced mitophagy in bronchial fibroblasts from severe asthmatic patients
Source: PLoS One. 2020 Nov 30;15(11):e0242695. doi: 10.1371/journal.pone.0242695 (PMC7704010; doi:10.1371/journal.pone.0242695)
Supplement: Attachment — Submitted filename: Response to Reviewers 2.docx [file pone.0242695.s002.docx]

Date: Aug 25 2020 02:35AM

To: "Qutayba Hamid" qalheialy@sharjah.ac.ae

From: "PLOS ONE" plosone@plos.org

Subject: PLOS ONE: Your submission PONE-D-20-12146R1

=============================================

IMPORTANT: PLEASE DO NOT REPLY TO THIS EMAIL

If you are unable to complete any points that are requested in this email, please explain why in the "Enter Comments" tab of the online submission form prior to re-submitting your manuscript. This will enable us to promptly assess your response and progress your manuscript to an Academic Editor at the earliest opportunity.

=============================================

PONE-D-20-12146R1

Enhanced mitophagy in bronchial fibroblasts from severe asthmatic patients

Dr. Qutayba Hamid

Dear Dr. Hamid,

Thank you for submitting your manuscript entitled "Enhanced mitophagy in bronchial fibroblasts from severe asthmatic patients" to PLOS ONE. Your manuscript files have been checked in-house but before we can proceed we need you to address the following issues:

1) Please upload a new copy of Figures 1, 2, 3 and 6 as the detail is not clear. Please follow the link for more information:

<https://blogs.plos.org/plos/2019/06/looking-good-tips-for-creating-your-plos-figures-graphics/>

Thank you so much for your comments and for bringing this to our attention. We hope we have addressed these concerns appropriately. We have improved the scaling and sizing of our images to improve its details.

2) It appears that your ORCiD iD has not been validated in your Editorial Manager account and we are unable to proceed until that step is complete.

To validate your ORCiD iD in Editorial Manager, please follow the steps below:

i. In your Editorial Manager account, please go to ‘Update my Information’ (in the upper left-hand corner of the main menu), and click on the Fetch/Validate link next to the ORCiD field.

ii. This link will take you to the ORCiD site and allow you to create a new iD or authenticate a pre-existing iD in Editorial Manager.

For additional instructions, please watch the following video for a step-by-step demonstration:

<https://www.youtube.com/watch?v=_xcclfuvtxQ>

The ORCID iD has been updated in the Editorial Manager account. It is “0000-0002-1107-190X”.

Your manuscript has been returned to your account. Please log on to PLOS Editorial Manager at

https://www.editorialmanager.com/pone/ to access your manuscript.

Your manuscript can be found in the "Revisions Sent Back to the Author" link under the New Submissions menu. After you have made the changes requested above, please be sure to view and approve the revised PDF after rebuilding the PDF to complete the resubmission process.

Please note that these changes have been requested to comply with submission guidelines and your manuscript will *not* be sent to review until you have fully adhered to our requests. Once your paper has been seen by an Editor we may return it to you for further information or amendments.

We ask that you address this request within 28 days. If you require additional time, please email the journal office. We are happy to grant extensions of up to one month past this due date. If we have not heard from you within 28 days, your manuscript will be withdrawn from Editorial Manager.

Thank you for submitting your work to PLOS ONE.

Kind regards,

Juliet MacAskill

PLOS ONE

__________________________________________________

In compliance with data protection regulations, you may request that we remove your personal registration details at any

time. (Use the following URL: https://www.editorialmanager.com/pone/login.asp?a=r). Please contact the publication

office if you have any questions.
