# Supplementary material for: Enhanced mitophagy in bronchial fibroblasts from severe asthmatic patients
Source: PLoS One. 2020 Nov 30;15(11):e0242695. doi: 10.1371/journal.pone.0242695 (PMC7704010; doi:10.1371/journal.pone.0242695)
Supplement: Attachment — Submitted filename: Response to Reviewers 3.docx [file pone.0242695.s003.docx]

Date: Oct 14 2020 06:23AM

To: "Qutayba Hamid" qalheialy@sharjah.ac.ae

From: "PLOS ONE" plosone@plos.org

Subject: PLOS ONE Decision: Revision required [PONE-D-20-12146R1]

PONE-D-20-12146R1

Enhanced mitophagy in bronchial fibroblasts from severe asthmatic patients

PLOS ONE

Dear Dr. Hamid,

Thank you for submitting your manuscript to PLOS ONE. After careful consideration, we feel that it has merit but does not fully meet PLOS ONE’s publication criteria as it currently stands. Therefore, we invite you to submit a revised version of the manuscript that addresses the points raised during the review process.

==============================

ACADEMIC EDITOR: While the study has been improved, the interpretation of p62 data needs additional attention. Namely, the unexpected finding that p62 levels were not increased in the presence of pepstatin/E64 could be explained by the relatively short incubation time (6 h). Also, the further increase in p62 levels in the presence of lysosomal proteolysis inhibitors indicates its transcriptional upregulation, which is consistent with its role in delivering autophagic cargo. The authors should briefly address these issues.

We would like to thank the Academic Editor for his comments. We have addressed these points appropriately in lines 533 and 551.

==============================

Please submit your revised manuscript by Nov 28 2020 11:59PM. If you will need more time than this to complete your revisions, please reply to this message or contact the journal office at plosone@plos.org. When you're ready to submit your revision, log on to https://www.editorialmanager.com/pone/ and select the 'Submissions Needing Revision' folder to locate your manuscript file.

Please include the following items when submitting your revised manuscript:

A rebuttal letter that responds to each point raised by the academic editor and reviewer(s). You should upload this letter as a separate file labeled 'Response to Reviewers'.

A marked-up copy of your manuscript that highlights changes made to the original version. You should upload this as a separate file labeled 'Revised Manuscript with Track Changes'.

An unmarked version of your revised paper without tracked changes. You should upload this as a separate file labeled 'Manuscript'.

If you would like to make changes to your financial disclosure, please include your updated statement in your cover letter. Guidelines for resubmitting your figure files are available below the reviewer comments at the end of this letter.

If applicable, we recommend that you deposit your laboratory protocols in protocols.io to enhance the reproducibility of your results. Protocols.io assigns your protocol its own identifier (DOI) so that it can be cited independently in the future. For instructions see:

http://journals.plos.org/plosone/s/submission-guidelines#loc-laboratory-protocols

We look forward to receiving your revised manuscript.

Kind regards,

Vladimir Trajkovic

Academic Editor

PLOS ONE
